# Association of mean arterial pressure with non-alcoholic fatty liver disease: results from the NAGALA study

**DOI:** 10.3389/fcvm.2023.1266879

**Published:** 2023-10-20

**Authors:** Xinghui Li, Huijian Yang, Guobo Xie, Maobin Kuang, Guotai Sheng, Yang Zou

**Affiliations:** ^1^ Department of Internal Medicine, Fuzhou Dongxiang District People’s Hospital, Fuzhou, China; ^2^ Department of Cardiology, Jiangxi Provincial People’s Hospital, The First Affiliated Hospital of Nanchang Medical College, Nanchang, China; ^3^ Jiangxi Cardiovascular Research Institute, Jiangxi Provincial People’s Hospital, The First Affiliated Hospital of Nanchang Medical College, Nanchang, China

**Keywords:** mean arterial blood pressure, MAP, non-alcoholic fatty liver disease, general population, NAGALA

## Abstract

**Objective:**

Several recent reports have suggested the use of mean arterial blood pressure (MAP) to assess/predict the risk of developing atherosclerosis, chronic kidney disease, diabetes, metabolic syndrome, and poor prognosis in a variety of cardiovascular and cerebrovascular diseases. The current study aimed to investigate the association of MAP with non-alcoholic fatty liver disease (NAFLD) and to explore the differences in this association across populations.

**Methods:**

This study used data from the NAGALA study from 1994 to 2016. MAP was calculated as 1/3 systolic blood pressure (SBP) + 2/3 diastolic blood pressure (DBP). Restricted cubic spline (RCS) and logistic regression models were used to examine the correlation of MAP with NAFLD.

**Results:**

The study population was 14,251 general people undergoing health screening, with a median (interquartile range) age of 42 (36–50) years; among them, 48% were women, and 2,507 (17.59%) were diagnosed with NAFLD. After fully controlling for confounders in the current dataset, MAP was positively and non-linearly associated with NAFLD [(odds ratios (ORs): 1.39, 95% confidence intervals (CIs): 1.15, 1.68); *P* for non-linearity = 0.024]; the dose-response curve showed that there was a transient saturation effect interval when MAP was between 85 and 95 mmHg, where the risk of NAFLD was neither increased nor decreased. The results of the stratified analysis showed that the risk of NAFLD associated with MAP appeared to be influenced only by age (*P*-interaction = 0.002), but not by sex, body mass index (BMI), habits of exercise, drinking status, or smoking status (*P*-interaction > 0.05); further age-stratified RCS analysis showed that the non-linear association between MAP and NAFLD in the young and middle-aged and the middle-aged and elderly populations was consistent with the results of the whole population, whereas, in the elderly population, a U-shaped curve association between MAP and NAFLD was observed, with both low and high MAP increasing the risk of NAFLD.

**Conclusion:**

In the general population, MAP was positively and non-linearly associated with NAFLD, and this association only differed significantly by age, but not by sex, BMI, habits of exercise, drinking status, and smoking status.

## Introduction

NAFLD is a global public health problem that affects billions of people worldwide (1, 2). In North America and Europe, the prevalence of NAFLD is 24%, in Asia, this figure is 27%, and in South America and the Middle East, the prevalence of NAFLD is as high as 31% and 32% (1, 3). Several recent predictive analyzes based on NAFLD Markov models showed that even if the prevalence of obesity and diabetes plateaus in the future, that of NAFLD will continue to grow globally until 2030 and the number of patients with cirrhosis and advanced liver disease will more than double due to an aging/increasing population (4, 5). Fortunately, early NAFLD is reversible, and improving awareness and diagnosis of NAFLD, as well as public health campaigns to promote healthy diet and exercise, can help control the future growth of the disease burden (4, 6, 7).

MAP, defined as the mean blood pressure throughout the cardiac cycle, is a major driver of vital organ perfusion (8, 9), reflecting vascular peripheral resistance and cardiac output (10), and has been widely used in the field of critical care, where 65 mmHg is considered a critical point of poor prognosis (11–15). In addition, the accuracy of using MAP to predict preeclampsia is better than SBP or DBP alone in early and mid-trimester pregnancies (16, 17). Together, these previous studies supported that MAP is sufficiently advantageous in short-term event assessment, and in recent years, MAP has begun to be mentioned in epidemiological studies; several researchers have found MAP to be of good value in assessing the risk of developing various chronic diseases such as atherosclerosis (18), diabetes (19–21), chronic kidney disease (22, 23), and metabolic syndrome (24), as well as in predicting poor prognosis in cardiovascular disease (9, 25–28). A series of epidemiological evidence further suggested that MAP may be a useful assessment tool for common chronic diseases; however, data on the direction of the association between MAP and the risk of developing NAFLD are still scarce, and it is unclear whether MAP is associated with NAFLD in the general population, whether this association is non-linear, and whether there are differences across populations. To clarify these issues, the current study aimed to assess the association of MAP with NAFLD in the general population based on large sample data from the NAGALA study.

## Methods

### Study design and participants

**Figure 1:**
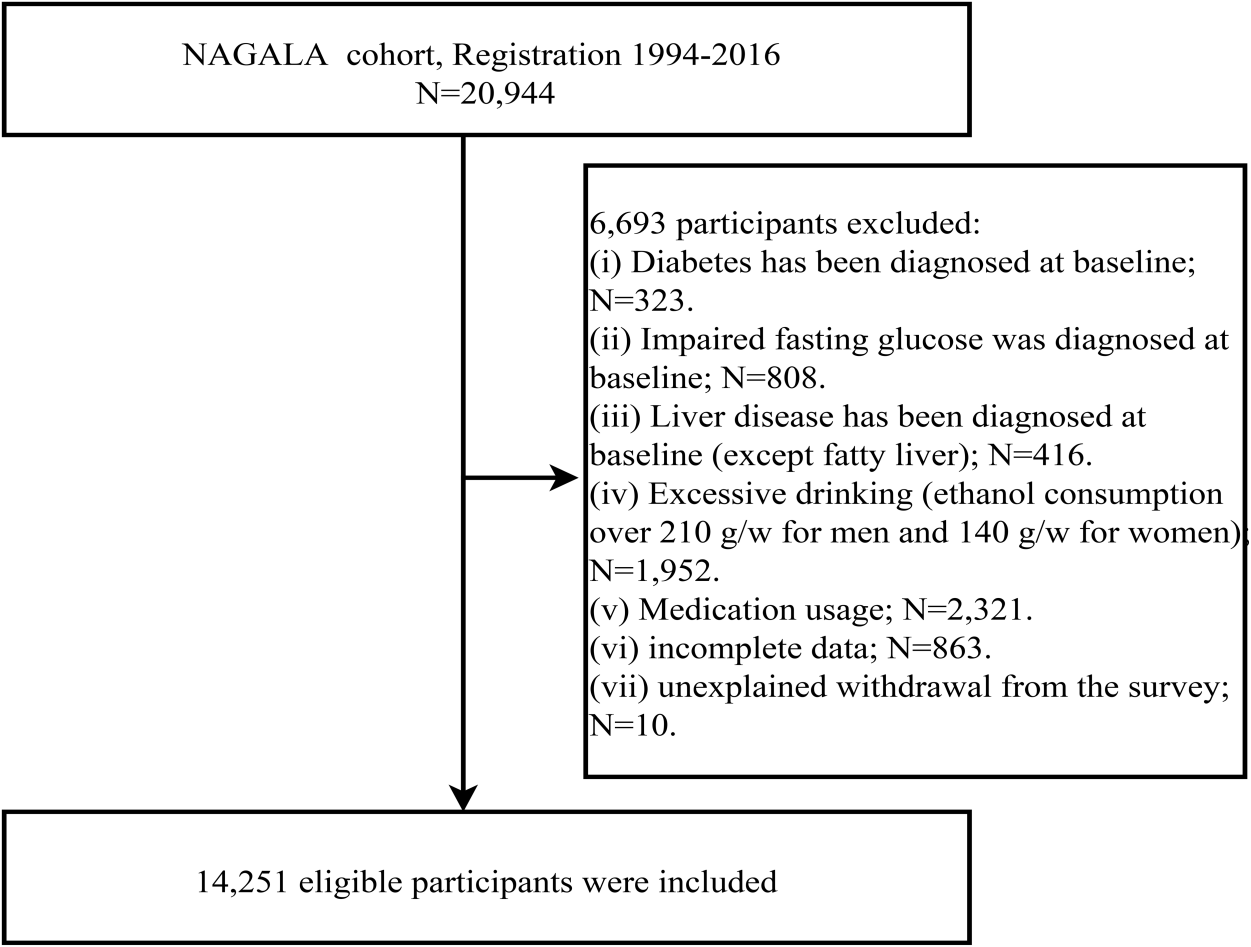
Flowchart of the selection process of study subjects.

We performed a post-hoc analysis of the data from the NAGALA study (the original data were obtained from the Murakami Memorial Hospital Physical Examination Center in Gifu, Japan, and the available data were uploaded to the Dryad database for public sharing) (29). NAGALA study was a longitudinal cohort study that continuously recruited 20,944 participants who received health checkups between 1994 and 2016 to assess risk factors for common chronic diseases with the aim of promoting public health, and it was authorized by the Ethics Committee of Murakami Memorial Hospital and informed consent was provided by all participants. The study design and part of the study results have been previously published (30). Based on the previous research, the current study used the de-identified public data in the NAGALA study for post-hoc analysis, aiming to analyze and explore the association between MAP and NAFLD in different populations. The new study protocol has been submitted to the Ethics Committee of Jiangxi Provincial People's Hospital for review and has been approved (IRB2021-066), and the STROBE reporting guidelines have been followed throughout the study. In addition, because the current data set has been de-identified, the Ethics Committee of Jiangxi Provincial People's Hospital exempted the informed consent of the subjects. According to the new study objectives we excluded participants with the following characteristics: (i) participants with diagnosed hepatitis (viral/alcoholic) or diabetes or fasting plasma glucose (FPG) above 6.1 mmol/L at baseline (*n* = 1,547); (ii) participants with alcohol intake ≥ 210 g/w for men or ≥ 140 g/w for women at baseline (*n* = 1,952) (31); (iii) participants who were taking medications at baseline (*n* = 2,321); (iv) participants with missing data at baseline (*n* = 863); and (v) participants who withdrew from the study (*n* = 10). Finally, we included 14,251 eligible participants, and the flowchart for selecting participants was shown in Figure 1.

### Data collection and measurement

Data on participants' demographic characteristics (sex, age), lifestyle (smoking and drinking status, habits of exercise), disease history, and medication history were recorded through face-to-face interviews using a standardized questionnaire. Height, weight, waist circumference, and blood pressure were measured using standard methods in a standard setting, and BMI was calculated as weight/height^2^. MAP was calculated as 1/3 SBP + 2/3 DBP (19). Smoking status was divided into non-smoking, former smoking, and current smoking; drinking status was divided into non, light, and moderate based on the amount and type of weekly alcohol consumption in the past month (32). Habits of exercise were evaluated based on the frequency of exercise per week, and those who exercised more than once were considered to have exercise habits. Venous blood specimens were drawn after participants had fasted for at least 8 h, and then glycosylated hemoglobin (HbA1c), alanine aminotransferase (ALT), high-density lipoprotein cholesterol (HDL-C), aspartate aminotransferase (AST), total cholesterol (TC), triglycerides (TG), and gamma-glutamyl transferase (GGT), FPG were measured using an automated biochemical analyzer according to standard procedures.

### Diagnosis of NAFLD

During the physical examination, the diagnosis of NAFLD was determined based on imaging; trained ultrasound technicians performed the abdominal ultrasound procedure, and then an experienced gastroenterologist reviewed the ultrasound images, scored them, and made the diagnosis of NAFLD based on the following four characteristics: hepatorenal echo contrast, liver brightness, deep attenuation and vascular blurring (33).

### Statistical analysis

Considering the large sample size included in this study, we proposed to characterize the data of participants by MAP quintiles. Baseline characteristics of participants were reported as mean with standard deviation or median with interquartile range, and comparisons between groups were made using one-way ANOVA or Kruskal-Wallis H test, depending on whether the data distribution was normal (assessed using Q-Q plots). Categorical data were then expressed as frequencies and percentages, and comparisons between groups were made using the chi-square test.

To analyze the association between MAP and NAFLD, we calculated the ORs and 95% CIs using logistic regression analysis; in addition, we calculated variance inflation factors (Supplementary Table S1) for checking potential multicollinearity for all covariates before modeling (34). Four stepwise adjusted models were constructed according to the methodology suggested by the STROBE statement (35). Model 1 was adjusted for age, BMI, and height; model 2 further considered the effects of smoking and drinking status and habits of exercise based on model 1; model 3 continued with adjustments for AST, ALT, and GGT based on model 2; model 4 was adjusted for all non-collinear variables as the final model, i.e., it additionally considered the effects of glycemic lipid factors (TG, HDL-C, TC, FPG, and HbA1c) based on model 3. We also assessed the dose-response relationship between MAP and NAFLD using the RCS on the basis of model 4, with nodes located at the 5th, 35th, 65th, and 95th percentiles of the exposure distribution (36).

To test the robustness of the association between MAP and NAFLD, we also performed 4 sensitivity analyses. Based on model 4, we performed the same analyses in the population without exercise habits, in the young and middle-aged population, in the population with normal blood pressure, and in the non-obese population, respectively.

To explore whether the association between MAP and NAFLD differed among different populations, we further performed stratified analyses according to age, sex, BMI, habits of exercise, smoking status, and drinking status, and used likelihood ratio tests to assess differences between different subgroups.

Data analysis was performed in November 2022 using R version 3.4.3 (The R Foundation for Statistical Computing) and Empower(R) version 2.0. Statistical tests were two-tailed, and *P* values less than 0.05 were considered statistically significant.

## Results

### Study subjects and characteristics

The study population was 14,251 general people undergoing health screening, with a median (interquartile range) age of 42 (36–50) years and 48% being women. Table 1 shows the baseline characteristics of participants across MAP quintiles. The results showed that the prevalence rates of NAFLD in the MAP quintiles were 3.75%, 9.82%, 15.37, 22.85 and 35.81%, respectively. The higher MAP group was older, had higher height, weight, BMI, and WC, more people who smoked and drank alcohol, and had higher levels of liver enzymes, blood sugar, blood pressure, and blood lipids except for HDL-C. Additionally, it is worth noting that with the MAP quartile increasing, the number of men gradually increased and the number of women gradually decreased.

**Table 1:** Baseline characteristics of four groups.

	MAP quintiles					
	Q1 (51.17–75.33)	Q2 (75.50–81.50)	Q3 (81.67–87.17)	Q4 (87.33–94.67)	Q5 (94.83–187.67)	*P*-value
No of subjects	2,830	2,841	2,810	2,902	2,868	
Sex						<0.001
Women	2,159 (76.29%)	1,613 (56.78%)	1,251 (44.52%)	1,016 (35.01%)	801 (27.93%)	
Men	671 (23.71%)	1,228 (43.22%)	1,559 (55.48%)	1,886 (64.99%)	2,067 (72.07%)	
Age, years	40.00 (35.00–46.00)	41.00 (36.00–48.00)	42.00 (37.00–50.00)	43.00 (37.00–51.00)	46.00 (39.00–53.00)	<0.001
Height, cm	161.41 (7.46)	164.04 (8.35)	165.53 (8.53)	166.35 (8.55)	166.60 (8.39)	<0.001
Weight, kg	51.60 (46.90–57.20)	55.80 (49.60–63.50)	60.20 (52.70–67.20)	63.10 (55.60–70.70)	66.60 (58.80–74.93)	<0.001
BMI, kg/m^2^	20.16 (2.21)	21.10 (2.51)	21.93 (2.64)	22.90 (3.02)	24.18 (3.48)	<0.001
WC, cm	70.17 (7.04)	73.53 (7.66)	76.10 (7.96)	78.77 (8.47)	82.21 (9.17)	<0.001
ALT, IU/L	14.00 (11.00–18.00)	15.00 (12.00–20.00)	16.00 (13.00–22.00)	19.00 (14.00–25.00)	20.00 (15.00–29.00)	<0.001
AST, IU/L	16.00 (13.00–19.00)	16.00 (13.00–20.00)	17.00 (14.00–20.00)	18.00 (15.00–22.00)	19.00 (15.75–23.00)	<0.001
GGT, IU/L	12.00 (10.00–15.00)	13.00 (11.00–18.00)	15.00 (11.00–20.00)	16.00 (13.00–24.00)	19.00 (14.00–29.00)	<0.001
HDL-C, mmol/L	1.58 (1.32–1.85)	1.47 (1.23–1.75)	1.40 (1.15–1.69)	1.36 (1.13–1.62)	1.28 (1.08–1.55)	<0.001
TC, mmol/L	4.81 (4.29–5.43)	4.94 (4.40–5.53)	5.07 (4.53–5.66)	5.20 (4.63–5.77)	5.33 (4.76–5.92)	<0.001
TG, mmol/L	0.54 (0.38–0.77)	0.63 (0.44–0.91)	0.73 (0.51–1.08)	0.82 (0.55–1.21)	0.98 (0.67–1.48)	<0.001
FPG, mmol/L	4.93 (0.38)	5.07 (0.40)	5.16 (0.39)	5.23 (0.39)	5.35 (0.38)	<0.001
HbA1c, %	5.13 (0.31)	5.15 (0.31)	5.17 (0.32)	5.20 (0.32)	5.23 (0.33)	<0.001
SBP, mmHg	95.78 (5.98)	105.35 (4.38)	112.54 (4.26)	120.56 (4.58)	135.01 (10.47)	<0.001
DBP, mmHg	57.84 (4.09)	65.20 (2.47)	70.31 (2.39)	75.87 (2.67)	86.08 (6.67)	<0.001
Habits of exercise	477 (16.86%)	469 (16.51%)	513 (18.26%)	513 (17.68%)	498 (17.36%)	0.446
Drinking status						<0.001
Non	2,566 (90.67%)	2,452 (86.31%)	2,315 (82.38%)	2,306 (79.46%)	2,166 (75.52%)	
Light	212 (7.49%)	293 (10.31%)	364 (12.95%)	423 (14.58%)	466 (16.25%)	
Moderate	52 (1.84%)	96 (3.38%)	131 (4.66%)	173 (5.96%)	236 (8.23%)	
Smoking status						<0.001
Non	2,064 (72.93%)	1,859 (65.43%)	1,684 (59.93%)	1,598 (55.07%)	1,541 (53.73%)	
Former	299 (10.57%)	387 (13.62%)	502 (17.86%)	651 (22.43%)	720 (25.10%)	
Current	467 (16.50%)	595 (20.94%)	624 (22.21%)	653 (22.50%)	607 (21.16%)	
NAFLD	106 (3.75%)	279 (9.82%)	432 (15.37%)	663 (22.85%)	1,027 (35.81%)	<0.001

Values were expressed as mean (standard deviation) or medians (quartile interval) or *n* (%). NAFLD, non-alcoholic fatty liver disease; BMI, body mass index; WC, waist circumference; ALT, alanine aminotransferase; AST, aspartate aminotransferase; GGT, gamma-glutamyl transferase; HDL-C, high-density lipoprotein cholesterol; TC, total cholesterol; TG, triglyceride; LDL-C, low density lipoprotein cholesterol; HbA1c, hemoglobin A1c; FPG, fasting plasma glucose; SBP, systolic blood pressure; DBP, diastolic blood pressure; MAP, mean arterial pressure.

### Multivariate regression model analysis of the association between MAP and NAFLD

At baseline, 2,507 (17.59%) participants were diagnosed with NAFLD. In the multivariate logistic analysis (Table 2), after gradually adjusting for potential confounders, we found that MAP and NAFLD always maintained a stable positive correlation, with a large OR variation between the highest MAP quintile and the other quintiles; overall, a positive correlation trend was maintained between MAP and NAFLD in all four models. In model 4, the risk of NAFLD increased by 15% for every 10 mmHg increase in MAP, and the risk of NAFLD was 1.75 times higher in those with MAP at greater than or equal to 94.83 mmHg (Q5) than in those with MAP at 51.17–75.33 mmHg (Q1).

**Table 2:** Logistic regression analyses for the association between MAP and NAFLD.

	Odds ratios (95% confidence interval)			
	Model 1	Model 2	Model 3	Model 4
MAP (Per 10mmHg increase)	1.18 (1.13, 1.25)	1.20 (1.14, 1.26)	1.20 (1.14, 1.27)	1.15 (1.09, 1.22)
Quintile 1	Ref	Ref	Ref	Ref
Quintile 2	1.50 (1.17, 1.93)	1.47 (1.14, 1.88)	1.57 (1.21, 2.04)	1.46 (1.12, 1.91)
Quintile 3	1.67 (1.31, 2.12)	1.66 (1.30, 2.11)	1.69 (1.31, 2.18)	1.48 (1.14, 1.91)
Quintile 4	1.70 (1.34, 2.16)	1.71 (1.34, 2.16)	1.72 (1.34, 2.20)	1.50 (1.16, 1.94)
Quintile 5	2.04 (1.61, 2.58)	2.07 (1.63, 2.62)	2.11 (1.64, 2.71)	1.75 (1.35, 2.27)
*P*-trend	<0.001	<0.001	<0.001	<0.001

Model 1 adjusted for age, BMI and height.

Model 2 adjusted for age, sex, BMI, height, habits of exercise, smoking status and drinking status.

Model 3 adjusted for age, sex, BMI, height, habits of exercise, smoking status, drinking status, ALT, AST and GGT.

Model 4 adjusted for age, sex, BMI, height, habits of exercise, smoking status, drinking status, ALT, AST GGT, TG, HDL-C, TC, FPG and HbA1c.

### Sensitivity analysis

Table 3 shows the results of the association between MAP and NAFLD in multiple relatively low-risk populations, and the four sensitivity analyses based on model 4 provided similar findings to the main analysis, which underscored the robustness of the current study results.

**Table 3:** Adjusted odds ratios and 95% confidence intervals for NAFLD risk associated with the MAP in different test populations: sensitivity analysis.

		MAP quintiles						
	No.of subjects	MAP (per 10 increase)	Quintile 1	Quintile 2	Quintile 3	Quintile 4	Quintile 5	*P*-trend
Sensitivity-1	11,774	1.17 (1.10, 1.25)	Ref	1.41 (1.05, 1.90)	1.42 (1.07, 1.89)	1.50 (1.13, 1.99)	1.80 (1.36, 2.39)	<0.001
Sensitivity-2	13,607	1.19 (1.12, 1.26)	Ref	1.52 (1.15, 2.00)	1.60 (1.23, 2.09)	1.66 (1.27, 2.15)	1.99 (1.53, 2.59)	<0.0001
Sensitivity-3	13,766	1.15 (1.07, 1.23)	Ref	1.45 (1.11, 1.90)	1.47 (1.14, 1.91)	1.50 (1.16, 1.94)	1.66 (1.28, 2.16)	0.002
Sensitivity-4	11,981	1.16 (1.08, 1.24)	Ref	1.34 (0.99, 1.81)	1.52 (1.14, 2.04)	1.42 (1.06, 1.90)	1.78 (1.32, 2.40)	<0.001

Adjusted for age, sex, BMI, height, habits of exercise, smoking status, drinking status, ALT, AST GGT, TG, HDL-C, TC, FPG and HbA1c.

(1) sensitivity-1: excluding subjects with exercise habits at baseline; (2) sensitivity-2: excluding subjects more than 60 years of age at baseline; (3) sensitivity-3: excluding subjects whose baseline SBP ≥ 140 mmHg or DBP ≥ 90 mmHg; (4) sensitivity-4: excluding subjects whose baseline BMI ≥ 25 kg/m^2^.

Habit of exercise was not included in model 4 of sensitivity-1; Age was not included in model 4 of sensitivity-2; BMI was not included in model 4 of sensitivity-4.

### Differences in the association between MAP and NAFLD across populations

We proceeded to assess the association between MAP and NAFLD in different subgroups of age, sex, BMI, habits of exercise, smoking status, and drinking status by stratified analysis and interaction tests (Table 4). We found that MAP-related NAFLD risk appeared to be associated only with age (*P*-interaction = 0.002), but not with sex, BMI, habits of exercise, drinking status, or smoking status (*P*-interaction > 0.05).

**Table 4:** Stratified associations between MAP and NAFLD by age, sex, BMI, habits of exercise, drinking status and smoking status.

Subgroup	Adjusted OR (95% CI)	*P*-value	*P-*interaction
Age (years)			0.002
18–44	1.30 (1.19, 1.41)	<0.0001	
45–59	1.07 (1.00, 1.16)	0.0650	
≥60	1.04 (0.82, 1.31)	0.7502	
Sex			0.601
Women	1.13 (1.02, 1.24)	0.0159	
Men	1.16 (1.09, 1.24)	<0.0001	
BMI (kg/m^2^)			0.098
<25	1.36 (1.27, 1.45)	<0.0001	
≥25	1.24 (1.13, 1.36)	<0.0001	
Habits of exercise			0.149
Yes	1.06 (0.93, 1.20)	0.4020	
No	1.17 (1.10, 1.24)	<0.0001	
Drinking status			0.391
Non	1.13 (1.06, 1.21)	<0.0001	
Light	1.19 (1.03, 1.38)	0.0178	
Moderate	1.30 (1.06, 1.61)	0.0141	
Smoking status			0.392
Non	1.17 (1.09, 1.27)	<0.0001	
Former	1.18 (1.05, 1.31)	0.0037	
Current	1.08 (0.96, 1.20)	0.1850	

CI, confidence interval; OR, odds ratios; other abbreviations as in Table 1.

Adjusted for age, sex, BMI, height, habits of exercise, smoking status, drinking status, ALT, AST GGT, TG, HDL-C, TC, FPG and HbA1c.

In each case, the model is not adjusted for the stratification variable.

### Non-linear analysis of MAP and NAFLD

**Figure 2:**
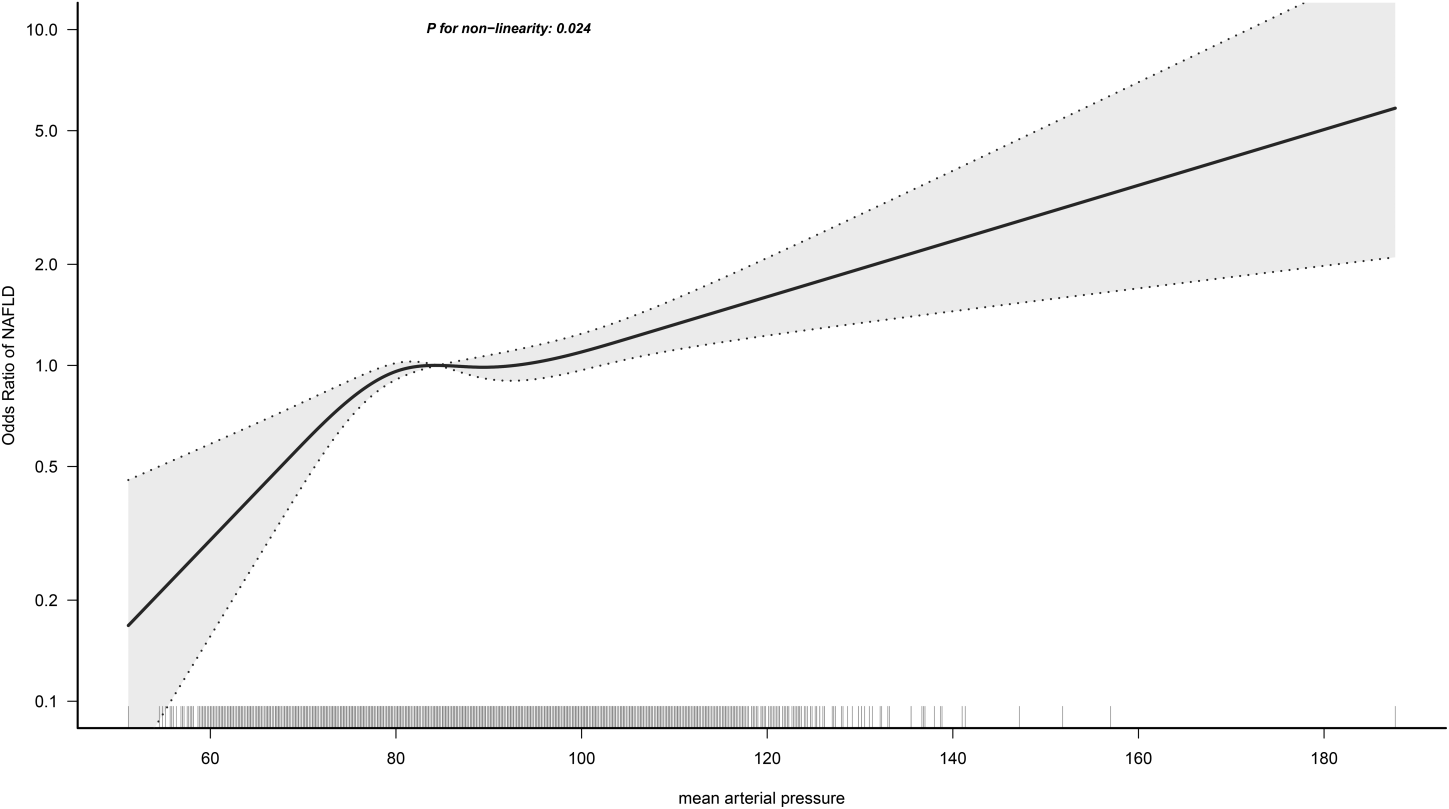
Dose-response curves for the association between MAP and NAFLD in the general population.

**Figure 3:**
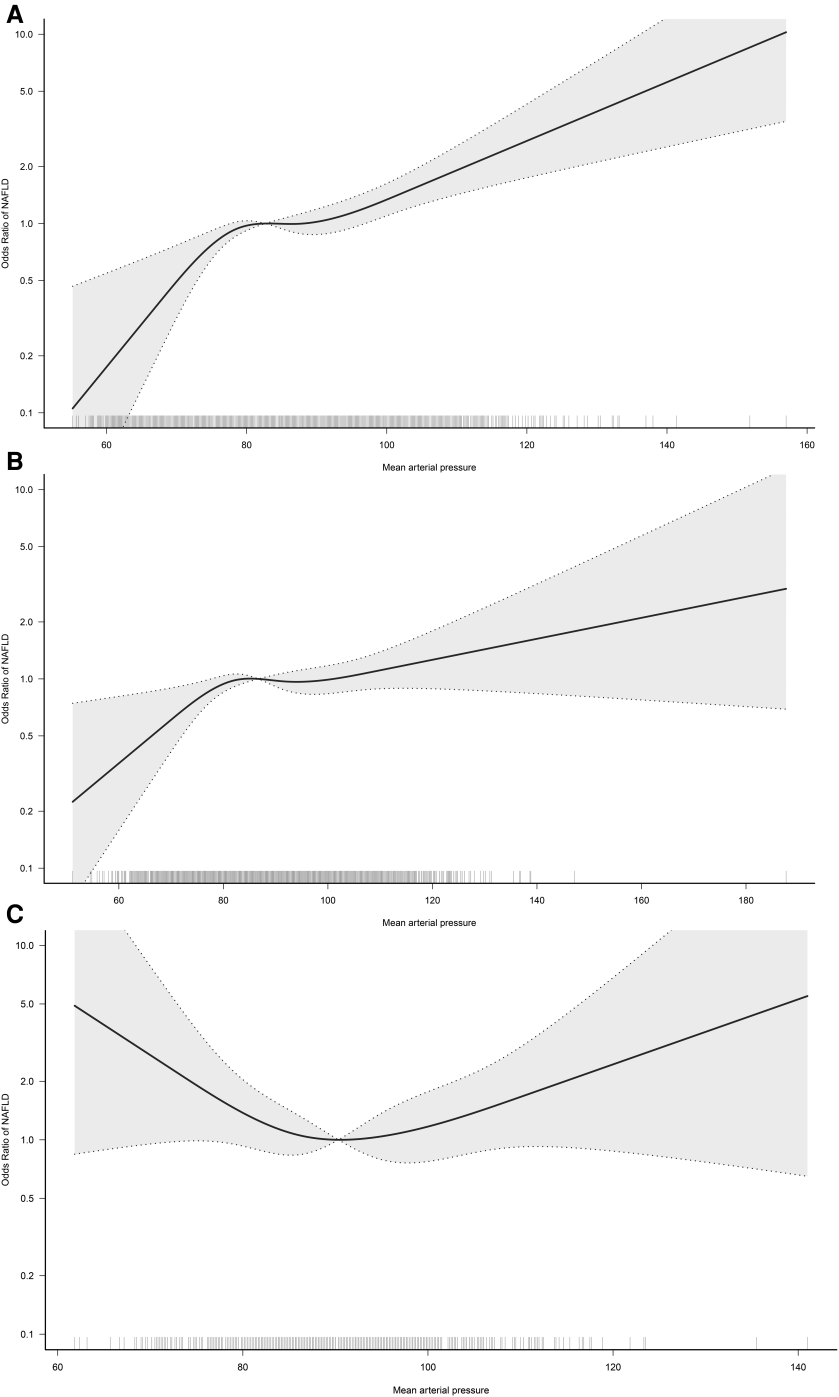
Dose-response curves for the association between MAP and NAFLD in 18–44 (**A**), 45–59 (**B**), and ≥60-year-old (**C**) populations. Adjusted for sex, BMI, height, habits of exercise, smoking status, drinking status, ALT, AST GGT, TG, HDL-C, TC, FPG and HbA1c.

In the RCS regression analysis, we more intuitively observed a positive association between MAP and NAFLD, although it is worth noting that the association between MAP and NAFLD was not straight linear (*P* for non-linearity = 0.024). As seen in Figure 2, in the general population, the risk of NAFLD neither increased nor decreased when MAP was between 85 and 95 mmHg, with a transient saturation effect interval, which was similar to the results in model 4 of Table 2 [OR: (Q3)1.48 vs. (Q4)1.50]. In addition, based on the results of stratified analysis, we continued to fit the dose-response curve of the association between MAP and NAFLD in different age groups (Figure 3); the results showed that the aforementioned saturation effect interval remained in the young and middle-aged population (18–44 years old) and in the middle-aged and elderly population (45–59 years old), while in the elderly population (≥60 years old), there was a U-shaped curve association between MAP and NAFLD, where the lowest point was about 90 mmHg, and both low and high MAP would increase the risk of NAFLD.

## Discussion

This study of 14,251 individuals aged 20–79 years in the general population provided evidence of an association between MAP and NAFLD, findings that may have implications for public health policy development. Our study found that MAP had a non-linear positive correlation with NAFLD, and when MAP was between 85 and 95 mmHg, the risk of NAFLD neither increased nor decreased, i.e., there was a transient saturation effect interval. After further RCS analysis according to age stratification, we found that this special saturation effect interval can also be observed in the young and middle-aged population and the middle-aged and elderly population, while in the elderly population, there was a U-shaped curve association between MAP and NAFLD, where the lowest point was approximately 90 mmHg, and both low and high MAP increased the risk of developing NAFLD.

The current study extended previous findings in several ways. First, the current study was the first to explore the association between MAP and NAFLD based on the general population and deeply assessed the differences in this association among different populations. To date, only one study has examined this relationship (37). In a January 2020 report by Xu and his colleagues, they reported for the first time that MAP was positively associated with NAFLD in non-obese women with normal low-density lipoprotein cholesterol levels, whereas this association was not present in non-obese men. In the current study, we also investigated the correlation between MAP and NAFLD in both sexes in subgroups. The results showed that MAP was independently associated with NAFLD in both sexes, and there was no significant difference in the risk of MAP-related NAFLD between the sexes (*P*-interaction = 0.601). In addition, we further evaluated the variation of the association between MAP and NAFLD in non-obese people and overweight/obese people. The results showed that although the risk of NAFLD related to MAP was relatively higher in non-obese people (OR: 1.36 vs. 1.24), the interaction test results suggested that no significant difference was found. Considering that the population surveyed in Xu et al.'s study has great particularity, the results of their study are limited in terms of extrapolation. In contrast, the present study was based on a general population and the results should be more reliable after implementing a rigorous statistical adjustment strategy and performing several sensitivity analyses.

Second, our study enriched the evidence for MAP in epidemiology. MAP is known to be defined as the mean blood pressure throughout the cardiac cycle and is a major driver of vital organ perfusion (8, 9). In a large number of early studies, MAP has been shown to be a reliable tool for assessing poor prognosis in critically ill patients and has been widely recommended in critical care medicine, surgery, and obstetrics and gynecology (11–17). And in recent years, MAP started to be mentioned in some epidemiological studies as well. Several studies pointed out that MAP was closely associated with diabetes (21) and was a predictor of cardiovascular events (38). More epidemiological investigations subsequently revealed that MAP can also be used to assess the risk of developing various chronic diseases such as atherosclerosis (18), chronic kidney disease (22, 23), and metabolic syndrome (24). All these research evidence supported that MAP not only has good application value in critical and severe cases but also should gradually receive attention in the epidemiological investigation. Our findings further enriched the references for the application of MAP in epidemics.

Third, the current study provided some useful information through further stratified analysis and RCS analysis. According to the results of the stratified analysis, we found that MAP-related NAFLD risk appeared to be influenced only by age (*P*-interaction = 0.002), but not by sex, BMI, exercise habits, drinking status, or smoking status (*P*-interaction > 0.05); in the results of the age-stratified analysis, the risk of MAP-associated NAFLD was significantly higher in the young and middle-aged population and the middle-aged and elderly population compared to the elderly population. The occurrence of this result is understandable in our opinion, because when age increases beyond 60 years, there is a gradual decrease in cardiac and vascular compliance and an increase in arterial wall stiffness, followed by a significant decrease in DBP (39, 40). While MAP is a composite of SBP and DBP, calculated as 1/3SBP + 2/3DBP (19), it can be seen that the contribution of DBP to the composition of MAP is greater. When DBP decreases significantly in older adults, MAP will also decrease significantly, and subsequently, MAP-related NAFLD risk is lower in data performance than in young and middle-aged populations. Similar findings were reported in a study by Wu et al. (19). In the study by Wu et al., they analyzed the association between MAP and diabetes and showed that the risk of MAP-related diabetes was progressively lower with increasing age (*P*-interaction < 0.001). Although these findings based on age stratification were similar, and even seemed to be somewhat explained by the evidence in the existing literature, considering the large blood pressure variability in the elderly population (41, 42), it may interfere with the study of the association between MAP and NAFLD in the elderly population. To address this issue, we further fitted the dose-response relationship curves between MAP and NAFLD in different age groups, and the results showed that in the young and middle-aged and the middle-aged and elderly populations, there would be a transient plateau in the risk of NAFLD when MAP was between 85 and 95 mmHg; while in the elderly population, there was a U-shaped curve association between MAP and NAFLD, with the lowest point being about 90 mmHg, and both low and high MAP would increase the risk of NAFLD. The above RCS analysis results provided new explanations for the prevention of NAFLD risk in different age groups. Combining the results of stratified analysis and RCS analysis, we suggested that it is appropriate to maintain MAP below 80–95 mmHg in the young and middle-aged population (25), while it may be appropriate to maintain MAP at 90 ± 10 mmHg in the elderly population.

### Study strengths and limitations

Several strengths of the current study are worth mentioning: first, MAP is a simple and easily understood measurement parameter, and for the first time we have identified the potential clinical use of this parameter in assessing NAFLD risk in the general population. Second, the current study assessed for the first time the non-linear association between MAP and NAFLD and the differences across different populations, and these findings provided useful reference material for subsequent studies and clinical applications. Third, the participants in the current study came from physical examination centers and had a large sample size, so the results of this study were more suitable for popularization among the general public, had good external applicability, and had a high reference value.

There are some limitations of the current study that need to be mentioned: The first and most important point was that the study cannot make a causal inference between MAP and NAFLD, because it was a cross-sectional design. Second, it is about the diagnosis of NAFLD. Combined with previous experience, the use of color Doppler ultrasound for the diagnosis of NAFLD may miss approximately 30% of patients with mild hepatic steatosis (43); in the current study, only 2,507 (17.59%) subjects were diagnosed with NAFLD at baseline, significantly lower than the global or Asian average prevalence of NAFLD (1, 3). It should be noted, however, that the current study analyzed the association between MAP and NAFLD at a much lower prevalence of NAFLD, which rather further validated the stability of the findings of the current study. Third, the current study population is from Japan, and regional and ethnic limitations exist (3, 44), and more studies based on different ethnicities and countries are needed. Fourth, as with all observational studies, the current study must inevitably be subject to residual confounding because the observed covariates are finite and the risk and protective factors are relatively infinite (45). However, the current study performed rigorous statistical adjustments as well as sensitivity analyses whenever possible, and the findings can be considered relatively reliable at this point. Fifth, calculating non-invasive fibrosis scores (such as NAFLD fibrosis score, Fibrosis-4 index, aspartate transaminase-to-platelet ratio index, etc.) to determine the characteristics of the patient cohort is highly beneficial for the current study in the absence of elastography imaging; However, due to the lack of some parameters for calculating the non-invasive fibrosis score in this dataset, further staging information of liver fibrosis could not be provided in the current study. Sixth, only patients with alcoholic fatty liver disease and viral hepatitis were excluded from the current study, which may lead to potential selection bias because there are many competing causes of liver disease, including autoimmune hepatitis, Wilson's disease, alpha-1-antitrypsin disease, and hemochromatosis.

## Conclusion

We observed a non-linear positive correlation between MAP and NALFD in the general population in a large NAGALA sample, where a transient saturation effect interval emerged for the risk of NAFLD when MAP was between 85 and 95 mmHg; while in the elderly population, there was a U-shaped curve association between MAP and NAFLD, with both low and high MAP increasing the risk of NAFLD. These new findings further enriched the evidence for MAP in epidemiology and provided new ideas for the prevention of NAFLD.

## Data Availability

The datasets presented in this study can be found in online repositories. The names of the repository/repositories and accession number(s) can be found in the article/**Supplementary Material**.
