# Supplementary material for: Association of mean arterial pressure with non-alcoholic fatty liver disease: results from the NAGALA study
Source: Front Cardiovasc Med. 2023 Oct 20;10:1266879. doi: 10.3389/fcvm.2023.1266879 (PMC10623437; doi:10.3389/fcvm.2023.1266879)
Supplement: Supplementary file 1 [file Table1.docx]

Supplementary Table 1: Collinearity diagnostics steps.

|  | VIF | | | | |
| --- | --- | --- | --- | --- | --- |
|  | Step 1 | Step 2 | Step 3 | Step 4 | Step 5 |
| MAP | Inf | 30.2 | 30.1 | 1.5 | 1.5 |
| Sex | 3.3 | 3.3 | 3.3 | 3.3 | 3.3 |
| Age | 1.4 | 1.4 | 1.4 | 1.4 | 1.3 |
| Weight | 168.5 | 168.5 | NA | NA | NA |
| Height | 51.9 | 51.9 | 2.9 | 2.9 | 2.4 |
| BMI | 95.9 | 95.9 | 5 | 5 | 1.7 |
| WC | 5.9 | 5.9 | 5.9 | 5.9 | NA |
| ALT | 4.1 | 4.1 | 4.1 | 4.1 | 4.1 |
| AST | 3.3 | 3.3 | 3.3 | 3.3 | 3.3 |
| GGT | 1.5 | 1.5 | 1.5 | 1.5 | 1.5 |
| Habits of exercise | 1 | 1 | 1 | 1 | 1 |
| HDL-C | 1.8 | 1.8 | 1.8 | 1.8 | 1.8 |
| TC | 1.5 | 1.5 | 1.5 | 1.5 | 1.5 |
| TG | 1.7 | 1.7 | 1.7 | 1.7 | 1.7 |
| FPG | 1.5 | 1.5 | 1.5 | 1.5 | 1.5 |
| HbA1c | 1.2 | 1.2 | 1.2 | 1.2 | 1.2 |
| Drinking status | 1.2 | 1.2 | 1.2 | 1.2 | 1.2 |
| Smoking status | 1.4 | 1.4 | 1.4 | 1.4 | 1.4 |
| SBP | Inf | NA | NA | NA | NA |
| DBP | Inf | 29.7 | 29.7 | NA | NA |

Abbreviations: Inf: infinity; VIF: Variance inflation factor; Other abbreviations as in Table ​1.

Note 1: VIF = 1/(1-R^2^).

Note-2: The variables with VIF >5 will be regarded as collinear variables and cannot be included in the multiple regression model.
